# Pilot study of separation surgery with intraoperative radiotherapy (IORT) for spine metastasis

**DOI:** 10.1016/j.jbo.2025.100737

**Published:** 2025-12-19

**Authors:** Baiyi Liu, Dongsheng Wang, Jian Zhang, Bo Huang, Mingying Geng, Peng Liu, Yaoyao Liu

**Affiliations:** a Department of Spine Surgery, Center of Orthopedics, Daping Hospital, Army Medical University, Chongqing, China; b Department of Radiotherapy Center, Daping Hospital, Army Medical University, Chongqing, China

**Keywords:** Spinal metastases, Spinal cord compression, Modified separation surgery, IORT

## Abstract

**Objective:**

This study aimed to introduce a novel modified separation surgery combined with intraoperative radiotherapy (MSS-IORT) treatment strategy for spinal metastasis and evaluate its efficacy and safety.

**Methods:**

A prospective study was conducted from January 2023 to June 2024. Patients with spinal metastasis exhibiting epidural spinal cord compression (ESCC) ≥ 2 grades and spinal instability neoplastic score (SINS) ≥ 7 were enrolled and underwent MSS-IORT. During the procedure, a dose of 8–10 Gy of IORT was administered to the tumor-invaded vertebrae segments during modified separation surgery. Pain intensity was assessed using the visual analog scale (VAS) preoperatively and at 1 week, 3 months, 6 months, and 12 months postoperatively. Neurological function was evaluated *via* the Frankel grade system, and functional status was measured using the Karnofsky performance scale (KPS) preoperatively and at 3, 6, and 12 months after surgery. Local control was evaluated based on X-ray, CT, or MRI examination. Survival time and perioperative complications were also documented.

**Results:**

A total of 38 patients (median age: 60 years) with 46 involved vertebrae were treated with MSS-IORT. The mean operation time was 277.5 min, and the mean blood loss was 750 ml. After a mean follow-up of 174.5 days, the VAS score decreased significantly postoperatively and continued to decline over time. The KPS score increased significantly at 6 and 12 months, and the Frankel grade significantly improved at 12 months. Local control failure occurred in 3 patients, and 13 experienced adverse events without IORT.

**Conclusion:**

The MSS-IORT strategy demonstrates both safety and efficacy, representing a promising treatment option for spine metastases, particularly in patients with ESCC grades ≥ 2 and SINS ≥ 7.

## Highlights

• MSS-IORT incorporating tumor resection, IORT, bone cement filling, and screw-rod fixation.
• MSS-IORT is a feasible and safe treatment option for spine metastasis patients with ESCC ≥ 2 and SINS ≥ 7.
• MSS-IORT effectively improves long-term local tumor control without increasing the incidence of complications.

## 1 Introduction

Advancements in cancer treatment have significantly improved patient survival time, resulting in a burgeoning population of metastatic patients. Bone is a common site for metastasis, particularly in prostate and breast cancer, with approximately 70 % of patients developing bone metastases [1]. Of these, spinal metastases (SM) are prevalent, accounting for 39 % of all bone metastases [2]. The presence of SM within the vertebral body, pedicles, and lamina has been demonstrated to result in spinal deformity and mechanical instability, which leads to potential for symptomatic spinal cord compression and severe pain, subsequently reducing the quality of life and survival time of patients [3].

Given the increasing life expectancy of SM patients, durable local control of spinal metastasis has become imperative [4]. Current therapeutic options for SM include spinal surgery, adjuvant radiation, and systemic therapy. In cases of unstable SM or severe deformities, surgical intervention can alleviate spinal cord compression by means of separating and excising epidural tumors, thereby safeguarding the spinal cord, facilitating radiotherapy around the cord [5], and restoring spinal stability. A range of surgical options is available, including separation surgery with postoperative radiotherapy, as well as total vertebral resection, etc. Given the high risks and complications associated with total vertebral resection, separation surgery combined with postoperative radiotherapy is preferred [6]. This approach aims to remove epidural lesions to decompress spinal cord compression, stabilize the spine, establish a histological diagnosis, and facilitate safe stereotactic body radiation therapy (SBRT) [4]. However, this approach does not fully decompress the spinal cord and necessitates a two-week postoperative wound healing period before SBRT treatment, delaying timely tumor control. IORT has emerged as an increasingly utilized treatment modality for cancers, including those of the breast, brain, and colon, over the past decades [7], [8], [9]. It can effectively shorten hospital stays, achieve excellent local control, and establish itself as a cost-effective and painless treatment strategy for breast cancer and brain metastasis [7], [10], [11]. IORT has been integrated into several international guidelines and is gaining growing popularity among clinicians worldwide[11]. IORT has also been explored as a potential strategy for treating stable SM [12]. For instance, the combination of kyphoplasty and IORT has shown effectiveness in alleviating pain and controlling local tumor progression in patients with stable vertebral SM [13]. The modification of separation surgery to ensure complete relief of spinal cord compression, in conjunction with IORT, has the potential to serve as an effective therapeutic strategy for the management of SM.

In this study, a modified separation surgery combined with intraoperative radiotherapy (MSS-IORT) treatment strategy was developed for SM patients with Epidural Spinal Cord Compression (ESCC) ≥ 2 grades and Spine Instability Neoplastic Score (SINS) ≥ 7. The approach entailed tumor curettage for spinal cord decompression, followed by IORT using the Intrabeam (ZEISS, Germany) system. Bone cement was employed to fill bone defects, while screws and rods were utilized for spinal fixation. A comprehensive evaluation of short- and long-term outcomes, encompassing neurological recovery, complications, and local tumor control, was conducted to furnish an innovative therapeutic alternative for SM patients with ESCC ≥ 2 and SINS ≥ 7.

## 2 Materials and methods

### 2.1 Patient data

**Fig. 1:**
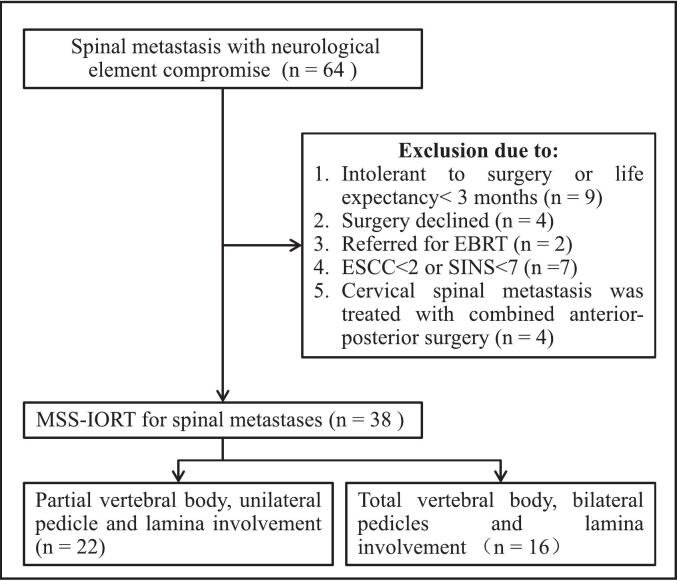
Flow diagram of patient enrollment and treatment for MSS-IORT.

A prospective study was conducted from January 2023 to June 2024 at our center, encompassing 64 patients with spinal cord compression resulting from SM. A total of 26 patients were excluded from the study, including 9 patients with poor health who could not tolerate surgery or had a life expectancy of less than 3 months based on the Tokuhashi score; 4 patients refused to undergo MSS-IORT treatment; 2 patients avoided surgery following a multidisciplinary team (MDT) consultation, as SBRT was considered sufficient to reduce lesions and alleviate symptoms; 7 patients with a ESCC＜2 or a SINS＜7 were also excluded from the study; and 4 patients with cervical metastatic tumors who were excluded to maintain homogeneity (Fig. 1). The remaining 38 patients underwent MSS-IORT treatment. Patient characteristics are delineated in Table 3, with comprehensive details provided in Supplementary Table S1. Ethical approval for this study was obtained from the Human Protection Committee at Army Medical University (Protocol No.2023–71), and the study was registered in the Chinese Clinical Trial Registry (ChiCTR2400088176) in adherence to the principles of the Declaration of Helsinki.

### 2.2 The inclusion criteria and exclusion criteria

Inclusion criteria: 1. Pathological examination to confirm the diagnosis of spinal metastases; 2. SINS score ≥ 7 and ESCC ≥ grade 2; 3. Patient accepted the MSS-IORT treatment and provided written informed consent.

Exclusion criteria. 1. Patients who could not tolerate surgery or who had a predicted survival time of < 3 Months were excluded. 2. ESCC < grade 2 or SINS < 7 points. 3. Reject the MSS-IORT treatment. 4. Patients with cervical metastases who underwent combined anterior and posterior surgeries were excluded.

### 2.3 Preoperative preparation

SM was diagnosed using a combination of X-ray, CT, magnetic resonance Imaging (MRI), and positron emission tomography (PET)-CT, along with the pathological diagnosis from needle biopsy samples. Preoperative evaluations were conducted in accordance with the NOMS framework, encompassing the assessment of cardiac, hepatic, renal, and neurological function, spinal integrity, and severity indexing utilising the Bilsky ESCC scale [14]. Spinal instability was analysed using the SINS, pain intensity was assessed *via* the visual analog scale (VAS), functional status was evaluated using the Karnofsky performance status (KPS) score, and spinal cord injury was assessed using the Frankel grade. The MDT, comprising a spinal tumor surgeon, medical oncologist, radiation oncologist, radiologist, anesthesiologist, dietitian, and vascular surgeon, executed the evaluation. The spine surgeon determined the surgical procedure. The medical oncologist was responsible for the systemic therapy of the tumor. The radiologist proceeds to interpret the radiographic data. The radiation oncologist delineated the IORT target zones and determined the optimal radiation dosage while the radiologist prepared the IORT equipment. The anesthesiologist assessed anesthesia risk. The dietician performed perioperative nutritional management. The vascular surgeon evaluated the necessity for preoperative arterial embolization, as well as embolization protocols, including abdominal aortic balloon block and/or highly selective embolization of the tumor’s feeding artery. The MDT meticulously devised perioperative treatment strategies.

### 2.4 The surgical and IORT procedure

**Fig. 2:**
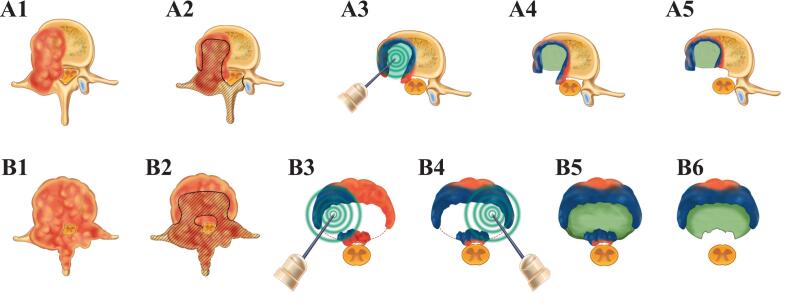
Schematic diagram of MSS-IORT procedures. A1-A5: MSS-IORT procedures for metastatic tumor invading the unilateral pedicle, lamina, and part of the vertebral body. (A1) Tumor invaded area; (A2) The resected area of tumor invaded pedicle, lamina, and vertebra; (A3) Schematic diagram of the IORT executed within the vertebra; (A4) Bone cement filled the residual cavity; (A5) Posterior vertebral wall was resected. B1-B5: MSS-IORT procedures for metastatic tumor invading the bilateral pedicles, lamina, and entire vertebral body. (B1) Tumor-invaded area; (B2) The resected area of tumor invaded pedicle, lamina, and vertebra; (B3-B4) Schematic diagram of the IORT executed within the left and right vertebra; (B5) Bone cement filled the residual cavity; (B6) Posterior vertebral wall was resected.

**Fig. 3:**
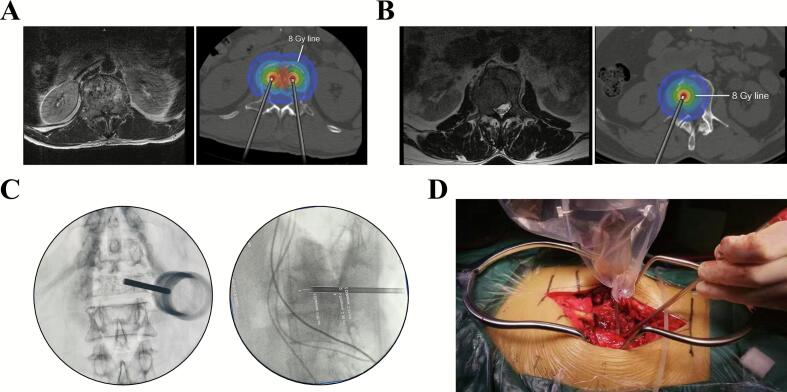
Procedure of MSS-IORT. (A) Preoperative planning for IORT for a tumor invading the bilateral pedicles, lamina, and entire vertebral body. (B) Preoperative planning for IORT for a tumor invading the unilateral pedicle, lamina, and part of the vertebral body. (C) The C-arm X-ray machine captured anteroposterior and lateral fluoroscopy of the applicator during the operation. (D) Intraoperative image of the IORT.

The MSS-IORT treatment strategy for MS is depicted in Fig. 2. Experienced spinal surgeons performed the surgical procedures, while a skilled radiologist administered IORT using the Intrabeam system. Initially, two sets of pedicle screws were implanted above and below the targeted vertebra. The posterior lamina and tumor were then excised from the dorsal spinal dura to achieve dorsal spinal cord decompression. For tumors invading the unilateral pedicle, lamina, and part of the vertebral body, the affected pedicle was removed. The vertebral tumor was subsequently scraped or drilled out as extensively as possible, while preserving the posterior vertebral cortes to minimize venous plexus bleeding and create a physical barrier for bone cement injection (Fig. 2A). In case of bilateral pedicles, lamina, and entire vertebral body involvement, bilateral pedicles were excised, and the vertebral body was partially removed, retaining the posterior vertebral cortex and one-third of the anterior vertebra (Fig. 2B). We place the applicator directly on the tumor bed at the pre-operation planned position (Fig. 3**A-B**) under direct visualization, a C-arm X-ray machine captured anteroposterior and lateral fluoroscopy was used to assist the applicator at this location (Fig. 3C). The radiologist then delivered IORT (Fig. 3D) with a therapeutic dose of 8–10 Gy [12], [15], ensuring both effective treatment and neurological safety. After tumor resection, the vertebral cavity was filled with bone cement. Once the bone cement had set, the invaded posterior vertebral cortex and residual tumor were carefully excised to achieve thorough ventral spinal cord decompression and nerve roots relaxation. Finally, long-segment fixation with screw‒rod instrumentation was performed, and the surgical incision was closed after complete hemostasis had been achieved.

### 2.5 Follow-up assessment

Post-surgical surveillance for patients was conducted at 1 week, 3 months, 6 months, and 12 months after surgery. The procedure entailed a comprehensive medical examination, systematic complication assessment, and neurological recovery evaluation. Local tumor control at the operative site was evaluated using radiological imaging techniques, including X-rays, CT scans, and MRI.

### 2.6 Statistical analysis

Data are presented as means ± standard errors of the means (SEMs). Statistical analyses were performed using GraphPad Prism 8.0. One-way ANOVA was used to compare the VAS, KPS, and Frankel scores. Patients were divided into 2 groups based on complications, and the *t*-test was employed to compare the scores between these groups. The Kaplan-Meier method was used to estimate local progression-free survival (L-PFS). A *p-*value of < 0.05 was considered statistically significant.

## 3 Results

The present study involved 38 patients (12 women, 26 men) with a median age of 60 years (range: 40–78) who underwent MSS-IORT treatment. The mean operation time was 277.5 min (range: 135–540), approximately 40 min longer than traditional separation surgery at our institution but 127.5 min shorter than previously reported separation surgery [16]. The mean blood loss was 750 ml, about 80 ml greater than that of traditional separation surgery at our hospital, but 33 ml less than reported for conventional separation surgery [16]. Follow-up data were available for 31 patients, with 7 lost to follow-up. Among the 31 patients, 12 patients had follow-up durations > 1 year, 7 patients had follow-up durations > 6 months, 6 patients had follow-up durations > 3 months, and 6 patients had follow-up durations < 3 months. Of these 31 patients, 11 had died (4 died within 1 month, 5 died within 6 months, and 2 died after 1 year postoperatively).

### 3.1 Pain and functional improvement

**Fig. 4:**
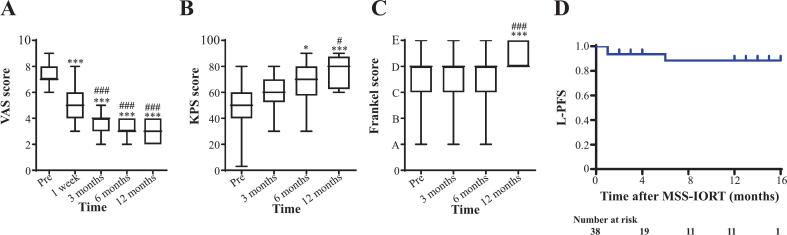
Patient outcomes after surgery. (A) VAS score before surgery (Pre), 1 week, 3, 6, and 12 months after surgery. (B-C) KPS score and Frankel grade before (Pre), 3, 6, and 12 months after surgery. (D) L-PFS of patients after MSS-IORT treatment. *p < 0.05, ^**^p < 0.01, ^***^p < 0.001*vs.* Pre group (A-C). ^###^*p* < 0.001 *vs.* 1-week group (A). ^#^*p* < 0.05, ^###^*p* < 0.001 *vs.* 3-month group (B & C).

The VAS scoring system utilizes a standard visual chart on a 0–10 scale to measure pain intensity, including in patients with spinal metastases [17]. The findings of the present study demonstrated a substantial decrease in the VAS score following surgery and continued to decline over time (Fig. 4A). This result suggests that pain is alleviated promptly postoperatively. The functional status of the patients was evaluated using the KPS score, with higher scores indicating better status. KPS scores demonstrated no significant change at 3 months post-surgery, but significantly increased at 6 and 12 months (Fig. 4B), suggesting functional improvement at 6 months and elevation over time. The degree of neural functional recovery was determined by evaluating the Frankel grade, which revealed no significant improvement at 3 and 6 months postoperatively; however, a significant advancement was observed at 12 months (Fig. 4C). These data indicate a slow but progressive neural functional recovery, which was confirmed at the 12-month follow-up.

### 3.2 Local and distant tumor control

In follow-up cases, one patient with SM from an epithelioid malignant neurinoma experienced recurrence at the spinal surgical site one month after MSS-IORT treatment and ultimately died approximately 2 months after surgery. Within a period of 3–6 months, two patients with spinal metastasis from lung cancer or bladder cancer experienced local recurrence. The patient with lung cancer who experienced recurrence was lost to follow-up for a further month, while the patient with bladder cancer continued to be observed. No local recurrence was detected in the remaining patients. The local progression-free survival (L-PFS) rates at 3, 6, and 12 months were 93.8 %, 88.8 %, and 88.8 %, respectively (Fig. 4D). In addition, the median follow-up time was 6 months.

### 3.3 Adverse events

Adverse events during surgery included impairment of the nerve root (1 patient) and dural tears with cerebrospinal fluid leakage (3 patients). Postoperative minor adverse events included pneumonia (1 patient), surgical site infection (1 patient), deep vein thrombosis (1 patient), poor wound healing (3 patients), and surgical site seroma (1 patient). Major postoperative adverse events included cerebral infarction (1 patient), autoimmune myocarditis (1 patient), worsening of general status (2 patients), and neurological decline (1 patient). In the latter case, preoperative spinal cord compression was severe, and postoperatively, lower extremity sensorimotor function decreased. Notably, no cases of radiation myelitis were observed (Table 1). Further analysis revealed that patients experiencing adverse events were significantly older (66.85 ± 5.93 years) than those without complications (60.60 ± 10.34 years) and had more blood loss, although the difference was not statistically significant (Table 2). No significant differences were identified in terms of surgery duration, screw utilization, SINS, KPS, or ESCC scores between the groups (Table 2).

**Table 1:** Adverse events.

**Adverse events**	**No. of Patients**
**Intraoperative**	
Nerve root injury	1
Dural laceration	3
**Postoperative (<30 days)**	
Minor	
Pneumonia	1
Surgical site infection	1
Deep venous thrombosis	1
Poor wound healing	3
Seroma	1
Major	
Cerebral infarction	1
Autoimmune myocarditis	1
Worsening of general status	2
Neurological worsen	1

**Table2:** Characteristics of patients experiencing adverse events.

	No AE (n = 25)	AE (n = 13)	*p*-value
Age (Y)	60.60 ± 10.34	66.85 ± 5.93	*P* = 0.024
Blood loss (ml)	688.00 ± 212.76	861.54 ± 347.70	*P* = 0.064
LOS (min)	303 ± 92	283 ± 67	*P* = 0.487
Screws (n)	7.12 ± 3.10	7.62 ± 2.63	*P* = 0.627
SINS (pt)	12[9], [10], [11], [12], [13], [14], [15], [16]	10[2], [3], [4], [5], [6], [7], [8], [9], [10], [11], [12], [13], [14]	*P* = 0.050
KPS	50[30–80]	60[30–80]	*P* = 0.863
ESCC	3[2], [3]	2[2], [3]	*P* = 0.091

Data is displayed as mean ± standard deviation or median [interquartile range].

Abbreviation: AE: Adverse event; ESCC, Epidural spinal cord compression scale; LOS, Length of surgery; min, Minutes; ns, non-significant；n, Number; pt, Points; SINS, Spinal neoplastic instability score; Y, Years;

### 3.4 Imaging follow-up examination

Among all the patients, 14 patients did not undergo imaging follow-up examination due to mortality, transition to hospice care, or loss of contact. A total of 24 patients underwent imaging follow-up after surgery, including X-ray, CT, or MRI examinations of the surgical site. Among them, 10 asymptomatic patients underwent X-ray examination alone. 14 patients underwent surgical site MRI or CT examination, and local recurrence was identified in 3 patients. No recurrence was detected in 11 patients, and 4 patients presented osteogenic enhancement signals on CT images from 6 to 12 months postoperatively (3 patients at 6 months and 1 patient at 12 months) (Supplementary Fig. S1).

## 4 Discussion

This study introduced a novel MSS-IORT approach for treating spinal metastasis in 38 patients at our center. Following MDT analysis, a personalized treatment plan was devised, incorporating tumor resection, IORT, bone cement filling, and screw-rod fixation. The VAS, Frankel, and KPS scores indicated that the treatment effectively mitigated pain and improved nerve function and functional status. In addition, the adverse event rates observed in this study were comparable to those reported in conventional separation surgery [5]. The long-term local control of the tumor was favorable. The findings of this study indicate that MSS-IORT is a safe and effective treatment strategy for patients with spinal metastasis who have an SINS score ≥ 7 and an ESCC score ≥ 2.

Tumor resection in this study adhered to the growth pattern of spinal metastasis (primarily two types) (Fig. 2). Compared with traditional separation surgery, lamina dissection has been demonstrated to expand the dorsal spinal cord space, thereby effectively alleviating compression. During the surgical procedure, the posterior vertebral wall was meticulously preserved and only removed after the bone cement injection, to minimize venous plexus bleeding and serve as a barrier for bone cement injection. The three spinal columns are of paramount importance for maintaining spinal stability. To achieve a balance between spinal stability and tumor resection, the anterior one-third of the vertebral body was preserved even tumor invaded. Furthermore, the vertebral cortex was preserved, thus facilitating the injection of bone cement without leakage. As a widely used orthopedic filler, bone cement is capable of effectively penetrating the bone trabecular space to fortify residual vertebral tissue. Simultaneously, the treatment produces heat to eradicate tumor cells and destroy sensory nerves to reduce bone cancer pain. In the present study, filling the tumor resection cavity with bone cement might be closely associated with increased vertebral stability, pain relief, and long-term local control.

The conventional approach of combining separation surgery with EBRT is a widely adopted treatment modality for spinal metastases [18]. However, population-based data indicate that a substantial proportion of patients never receive timely postoperative irradiation. Bhanot et al. reported that only 56 % of 1,730 spinal-metastasis patients underwent postoperative radiotherapy within 8 weeks after surgery [19], and Dugan JE et al. found that only 113 of 239 patients (47.3 %) who had undergone separation surgery received radiotherapy within 3 months postoperatively [20]. Delayed or omitted postoperative radiotherapy is associated with an increased incidence of radiographic local progression and worse oncological outcomes [21], [22], [23]. An additional drawback of conventional EBRT after separation surgery is the need to account for beam attenuation and scatter caused by metallic spinal instrumentation [24]. The Intrabeam system for IORT delivers low-energy X-rays that offer superior energy density and rapid dose attenuation, enabling direct targeting of the tumor bed while sparing adjacent tissues during the surgery session. This approach can eliminate the compliance gap associated with postponed EBRT [10]. Single-dose IORT is already established in breast cancer treatment, which directly administers radiotherapy to the tumor bed following breast tumor resection, significantly enhances patient compliance, and offers superior protection to adjacent healthy tissues [11]. Similarly, IORT for brain metastases shows a favorable toxicity profile and excellent local control, allowing an early transition to systemic therapy [7]. In the spine, Ali et al. reported that a dose of 8 Gy for local spinal metastasis is effective and safe [12]. while Ilya Laufer et al. discovered that doses of up to 10 Gy delivered to the spinal cord are safe, whereas doses exceeding 20 Gy may cause demyelination alterations [15]. Therefore, we used a needle applicator to emit spherical rays at the tumor center for optimal coverage while keeping the maximum spinal cord dose below 8 Gy [15]. IORT exhibits the characteristics of high energy at the center and rapid attenuation around the periphery. Based on the size and shape of the tumor in the vertebral body, the irradiation radius of the applicator and the treatment dose were determined before the operation. The dose at the edge of the irradiation radius is set at either 8 Gy or 10 Gy. The rationale behind these two dose levels is as follows: if the tumor shows signs of suspicious invasive growth at the outer edge of the set radius, a dose of 10 Gy is adopted; if there is no indication of tumor infiltration within the set radius, a dose of 8 Gy should be used.

The present study demonstrated that IORT treatment added only 10 min to operative time. Because the irradiation field is precisely confined to the tumor bed, MSS-IORT also spares adjacent radiosensitive structures such as the spinal cord and major blood vessels, thereby lowering radiation-related toxicity. No instances of radiation myelopathy were observed in the present study. Limitations of IORT should nevertheless be acknowledged. The dose distribution around the applicator is predictable, but may leave cold spots when tumors are large, irregular, or juxtaposed to critical vessels. To minimise the risk of local progression, we therefore recommend supplemental EBRT—typically delivered 2–4 weeks after wound healing—to eradicate possible residual tumor.

Regarding spinal body fixation, the screw-rod system was used to prevent spinal deformity and neurological decline in long-segment spinal body stabilization, thereby ensuring the securement of the upper and lower segments of the metastatic spine. Notably, no cases of postoperative spinal instability were observed in the present study.

Adverse events are prevalent in patients undergoing tumor surgery. The present study comprised 13 patients who experienced various adverse events, which were correlated with patient age and aligned with prior research. The surgical adverse event rate did not exceed that of traditional separation surgery [25]. The patients’ mean age (approximately 62.7 years) aligned with the typical age for conventional spinal metastasis surgery (average: 60 years) [26]. Although the evidence from earlier research indicates blood loss and surgery duration are strongly associated with adverse events [16], the present study did not observe a statistically significant increase in these parameters between patients who did and did not experience adverse events. This may be attributable to the limited sample size. It is noteworthy that the median operation time was 127.5 min shorter, and blood loss was 33 ml less than that of conventional separation surgery [16]. This partly elucidates the safety profile of this surgical strategy Table 3.

**Table 3:** Characteristics of the enrolled patients.

**Parameters**	**N(range)**	**%**
**Gender**		
Female	12	31.5 %
Male	26	68.5 %
**Age (median)**	65 (40–78)	
**KPS (initial) Primary tumors**		
80 ∼ 100	4	10.5 %
50 ∼ 70	23	60.6 %
<50	11	28.9 %
**ESCC grade**		
2	23	60.5 %
3	15	39.5 %
**Frankel score**		
A	3	7.9 %
B	2	5.2 %
C	14	36.8 %
D	16	42.1 %
E	3	7.9 %
**SINS category**		
Potentially unstable	22	57.8 %
Unstable	16	42.2 %
**Primary tumors**		
Lung cancer	21	55.3 %
Prostate cancer	4	10.5 %
Gastrointestinal cancer	4	10.5 %
Genitourinary cancer	4	10.5 %
Neurogenic malignant tumor	3	10.5 %
Thyroid cancer	2	5.2 %
**Disease spread**		
Single vertebra	11	28.9 %
2-4 vertebrae	15	39.4 %
Scattered	12	32.7 %
**Location**		
Thoracic spine	15	39.4 %
Lumbar spine	19	50 %
Sacrum	4	10.6 %
**Duration of neurologic symptoms before surgery**	35 (3 did not show neurological symptoms)	
<24 h	2	5.7 %
24-48 h	3	8.5 %
3-7 days	7	20 %
8-14 days	13	37.1 %
>14 days	10	28.7 %
**IORT dose**	46 vertebras	
8 Gy	20	43.5 %
10 Gy	26	56.5 %

Abbreviations: IORT, Intraoperative radiation therapy; SINS, spinal instability neoplastic score; ESCC, epidural spinal cord compression; KPS, Karnofsky performance scale.

Patients with progressive metastatic cancer require prompt, effective systemic treatment. For those with spinal instability and spinal cord compression, local treatment must be brief, ensure sustained tumor control, and provide instant vertebral stability. This combined surgical and radiation therapy approach offers rapid local tumor control. Our study demonstrated a rapid attenuation of VAS score, improved function, and reduced neurological interference, which may be attributable to the prompt spinal cord and nerve root decompression. In addition, bone cement injections and intraoperative radiotherapy may contribute to local tumor control and pain relief. A limitation of this study is encompassing various tumor types and postoperative systemic treatments. However, antihormone therapy or chemotherapy for prostate/breast cancer can facilitate local control, whereas patients with lung cancer tend to have a poor response [27]. Among 18 lung cancer patients, only 1 experienced local control failure. Additionally, 2 patients with bladder cancer or epithelioid malignant neurinomas experienced local control failure, which partially supported the effectiveness of the strategy. Our findings highlight the feasibility and rationale of MSS-IORT, warranting further investigation to reduce hospital stays and economic burden, which is significant for societal and economic development.

### 4.1 Limitations

This novel treatment approach has been applied to a limited number of patients, with a relatively short follow-up period, necessitating longer-term assessment to determine surgical efficacy.

## 5 Conclusion

We introduce MSS-IORT as a feasible and safe treatment option for SM patients with ESCC ≥ 2 and SINS ≥ 7. It effectively improves long-term local tumor control without increasing the incidence of complications. Nevertheless, additional research in broader populations and long-term periods is needed to determine its role in SM treatment.

## CRediT authorship contribution statement

**Baiyi Liu:** Writing – original draft. **Dongsheng Wang:** Writing – review & editing. **Jian Zhang:** Investigation. **Bo Huang:** Software. **Mingying Geng:** Funding acquisition. **Peng Liu:** Visualization, Conceptualization. **Yaoyao Liu:** Project administration.

## Funding

The author(s) disclosed receipt of the following financial support for the research, authorship, and/or publication of this article: This work was supported by the National Natural Science Foundation of China (81902257) and the Natural Science Foundation of Chongqing (2024TIAD-KJFZMSX0076).

**Data availability statement**

The data generated and/or analyzed during the current study are available from the corresponding author on reasonable request.

## Declaration of competing interest

The authors declare that they have no known competing financial interests or personal relationships that could have appeared to influence the work reported in this paper.

## References

[1] Fornetti J., Welm A.L., Stewart S.A. (2018). *Understanding the Bone in Cancer Metastasis*. J. Bone Miner. Res..

[2] Zhang H.R. (2020). *Percutaneous vertebral augmentation procedures in the management of spinal metastases*. Cancer Lett..

[3] Clezardin P. (2021). *Bone metastasis: mechanisms, therapies, and biomarkers*. Physiol. Rev..

[4] Spratt D.E. (2017). *An integrated multidisciplinary algorithm for the management of spinal metastases: an International Spine Oncology Consortium report*. Lancet Oncol..

[5] Li R.F. (2022). *Separation Surgery in the Treatment of Spinal Metastasis*. Technol. Cancer Res. Treat..

[6] Sakaura H. (2004). *Outcome of total en bloc spondylectomy for solitary metastasis of the thoracolumbar spine*. J. Spinal Disord. Tech..

[7] Dejonckheere C.S. (2025). *Intraoperative radiotherapy for resectable brain metastases: a systematic review and meta-analysis*. Radiother. Oncol..

[8] Harris E.E.R., Small W. (2017). *Intraoperative Radiotherapy for Breast Cancer*. Front. Oncol..

[9] Haddock M.G. (2017). *Intraoperative radiation therapy for colon and rectal cancers: a clinical review*. Radiat. Oncol..

[10] Dejonckheere C.S. (2023). *Intraoperative or postoperative stereotactic radiotherapy for brain metastases: time to systemic treatment onset and other patient-relevant outcomes*. J. Neurooncol.

[11] Das A. (2025). *The evolution of targeted intra operative radiotherapy in early breast cancer*. J. Cancer Res. Clin. Oncol..

[12] Ebad Ali S.M., Abbasi A.N., Zahoor N. (2022). *Outcomes of Intraoperative Radiotherapy (Iort) for Spinal Tumours*. J. Ayub Med. Coll. Abbottabad.

[13] Bludau F. (2020). *Long-term outcome after combined kyphoplasty and intraoperative radiotherapy (Kypho-IORT) for vertebral tumors*. Radiat. Oncol..

[14] Laufer I. (2013). *The NOMS framework: approach to the treatment of spinal metastatic tumors*. Oncologist.

[15] Laufer I. (2013). *Local disease control for spinal metastases following “separation surgery” and adjuvant hypofractionated or high-dose single-fraction stereotactic radiosurgery: outcome analysis in 186 patients*. J. Neurosurg. Spine.

[16] Echt M. (2021). *Separation surgery for metastatic epidural spinal cord compression: comparison of a minimally invasive versus open approach*. Neurosurg. Focus.

[17] Chen Q. (2021). *The emergence of new prognostic scores in lung cancer patients with spinal metastasis: a 12-year single-center retrospective study*. J. Cancer.

[18] Sciubba D.M. (2021). *Spinal metastases 2021: a review of the current state of the art and future directions*. Spine J..

[19] Bhanot K. (2022). *Survival after surgery for spinal metastases: a population-based study*. Can. J. Surg..

[20] Dugan J.E. (2024). *Obstacles to receiving postoperative radiation therapy following separation surgery for metastatic spine disease*. J. Neurosurg. Spine.

[21] Gong Y. (2021). *Delayed postoperative radiotherapy increases the incidence of radiographic local tumor progression before radiotherapy and leads to poor prognosis in spinal metastases*. Radiat. Oncol..

[22] Lee R.S. (2018). *Timing of surgery and radiotherapy in the management of metastatic spine disease: expert opinion*. J Spine Surg.

[23] Kumar N. (2020). *Is there an optimal timing between radiotherapy and surgery to reduce wound complications in metastatic spine disease?*. A Systematic Review. Eur Spine J.

[24] Li J. (2015). *Influence of internal fixation systems on radiation therapy for spinal tumor*. J. Appl. Clin. Med. Phys..

[25] Di Perna G. (2020). *Separation surgery for metastatic epidural spinal cord compression: a qualitative review*. J Bone Oncol.

[26] Wewel J.T., O'Toole J.E. (2020). *Epidemiology of spinal cord and column tumors*. Neurooncol Pract.

[27] Bauml J. (2013). Determinants of survival in advanced non–small-cell lung cancer in the era of targeted therapies. Clin. Lung Cancer.

