# Supplementary material for: Pilot study of separation surgery with intraoperative radiotherapy (IORT) for spine metastasis
Source: J Bone Oncol. 2025 Dec 19;56:100737. doi: 10.1016/j.jbo.2025.100737 (PMC12811523; doi:10.1016/j.jbo.2025.100737)
Supplement: Supplementary Data 1 [file mmc1.docx]

**Supplementary Table 1: Detailed characteristics of 38 patients**

| Patient | Age(y) | Entity | SINS | ESCC | Pre-KPS | Pre-Frankel | Pre-VAS | Vascular intervention | Proc | Blood loss (mL) | | Follow up(d) |  |
| --- | --- | --- | --- | --- | --- | --- | --- | --- | --- | --- | --- | --- | --- |
| 1 | 46 | Bladder cancer | 14 | 3 | 70 | C | 7 | NO | L2 L3 L4R L5 | | 500 | 686 |  |
| 2 | 65 | Breast cancer | 15 | 3 | 50 | C | 8 | NO | T9 T10 T11L L1 L2 | | 600 | 663 |  |
| 3 | 40 | Breast cancer | 13 | 2 | 80 | D | 6 | NO | L2 L3 L4L L5 S1 | | 500 | 659 |  |
| 4 | 57 | NSCLC | 13 | 2 | 80 | C | 8 | NO | L3 L4 L5L S1 | | 500 | 655 |  |
| 5 | 66 | Hepatocellular carcinoma | 12 | 3 | 50 | C | 9 | NO | T4 T5 T7 T8 | | 400 | 630 |  |
| 6 | 50 | Chordoma | 9 | 3 | 80 | D | 5 | YES | NO | | 700 | 626 |  |
| 7 | 60 | NSCLC | 12 | 2 | 60 | D | 7 | YES | T3 T4 T6 T7 | | 1100 | 623 |  |
| 8 | 65 | NSCLC | 14 | 2 | 50 | D | 8 | NO | T10 T11 L1 L2 | | 1000 | 514 |  |
| 9 | 70 | NSCLC | 13 | 2 | 70 | D | 7 | NO | T12 L1 L3 L4L | | 700 | 486 |  |
| 10 | 60 | NSCLC | 9 | 2 | 70 | D | 6 | NO | L4 L5 S1 | | 800 | 420 |  |
| 11 | 73 | NSCLC | 14 | 3 | 30 | C | 9 | YES | L4 L5 S1 S2 | | 1100 | 358 |  |
| 12 | 78 | NSCLC | 10 | 3 | 30 | B | 8 | NO | T8 T9 T11 T12 | | 1000 | 347 |  |
| 13 | 51 | Bladder cancer | 12 | 2 | 60 | D | 8 | NO | T12 L1 L2L L3 L4 | | 1000 | 233 |  |
| 14 | 75 | NSCLC | 15 | 3 | 40 | D | 8 | NO | L1 L2 L3R L4 L5 | | 800 | 185 |  |
| 15 | 60 | NSCLC | 9 | 2 | 80 | E | 7 | NO | T7 T8 T10 T11 | | 400 | 180 |  |
| 16 | 49 | Prostate cancer | 16 | 3 | 40 | C | 7 | NO | T3 T4 T6 T7 | | 1000 | 178 |  |
| 17 | 66 | Hepatocellular carcinoma | 11 | 3 | 30 | A | 7 | NO | T8 T9 T11 T12 | | 800 | 171 |  |
| 18 | 56 | Renal cell cancer | 11 | 2 | 60 | D | 8 | YES | L3 L4 L5R S1 S2AI | | 500 | 169 |  |
| 19 | 61 | Chordoma | 9 | 3 | 80 | D | 7 | YES | NO | | 800 | 148 |  |
| 20 | 69 | Renal cell cancer | 10 | 2 | 50 | D | 8 | YES | L1 L2 L3L L4 L5 | | 800 | 137 |  |
| 21 | 72 | Prostate cancer | 14 | 2 | 50 | D | 7 | YES | T7 T8 T9 T11 T12 | | 700 | 129 |  |
| 22 | 69 | NSCLC | 10 | 2 | 50 | D | 7 | NO | L1 L2R L3 L4 | | 400 | 121 |  |
| 23 | 48 | NSCLC | 14 | 2 | 60 | D | 7 | NO | T11 T12 L2 L3 | | 800 | 102 |  |
| 24 | 72 | Prostate cancer | 14 | 2 | 50 | D | 7 | NO | T11 T12 L2 L3 | | 600 | 95 |  |
| 25 | 77 | Colorectal cancer | 8 | 2 | 60 | D | 7 | NO | NO | | 400 | 93 |  |
| 26 | 60 | NSCLC | 8 | 2 | 60 | D | 8 | NO | L2 L3 L4R L5 S1 | | 800 | 64 |  |
| 27 | 58 | NSCLC | 10 | 2 | 60 | D | 8 | YES | T11 T12L L1 L2L L3 L4 | | 900 | 64 |  |
| 28 | 49 | NSCLC | 12 | 3 | 40 | C | 8 | NO | L3 L4 L5R S1 | | 600 | 30 |  |
| 29 | 72 | NSCLC | 14 | 3 | 30 | C | 9 | NO | T11 T12 L1L L3 L4 | | 1400 | 24 |  |
| 30 | 67 | Malignant neurinoma | 13 | 2 | 80 | E | 8 | NO | C7 T1 T3 T4 | | 900 | 22 |  |
| 31 | 67 | NSCLC | 11 | 3 | 30 | A | 7 | NO | T1 T2 T4 T5 T6 | | 900 | 16 |  |
| 32 | 71 | NSCLC | 10 | 2 | 40 | B | 6 | NO | T1 T2 T4 T5 T6 | | 1500 | 14 |  |
| 33 | 73 | Colorectal cancer | 11 | 2 | 70 | D | 8 | NO | NO | | 600 | LTF |  |
| 34 | 70 | Prostate cancer | 10 | 3 | 40 | C | 7 | NO | T9 T10R T12 L1 | | 400 | LTF |  |
| 35 | 65 | NSCLC | 9 | 2 | 70 | D | 7 | NO | L3 L4R L5 S1 | | 1000 | LTF |  |
| 36 | 65 | NSCLC | 14 | 2 | 60 | D | 7 | NO | L4 L5R S1 | | 600 | LTF |  |
| 37 | 53 | NSCLC | 12 | 3 | 60 | E | 8 | NO | T6 T7 T9 T10 | | 500 | LTF |  |
| 38 | 59 | NSCLC | 14 | 3 | 30 | A | 7 | NO | C5 C6 C7 T3 T4 T5 | | 400 | LTF |  |
| Abbreviation: NSCLC, nonsmall-cell lung cancer, LTF, loss-to-follow up. | | | | | | | | | | | | | |
